# Population Genetics of *Ceratitis capitata* in South Africa: Implications for Dispersal and Pest Management

**DOI:** 10.1371/journal.pone.0054281

**Published:** 2013-01-16

**Authors:** Minette Karsten, Bettine Jansen van Vuuren, Adeline Barnaud, John S. Terblanche

**Affiliations:** 1 Evolutionary Genomics Group, Department of Botany and Zoology, University of Stellenbosch, Matieland, South Africa; 2 Centre for Invasion Biology, Department of Zoology, University of Johannesburg, Auckland Park, South Africa; 3 IRD, Montpellier, France; 4 Centre for Invasion Biology, Department of Conservation Ecology and Entomology, Stellenbosch University, University of Stellenbosch, Matieland, South Africa; North Carolina State University, United States of America

## Abstract

The invasive Mediterranean fruit fly (medfly), *Ceratitis capitata*, is one of the major agricultural and economical pests globally. Understanding invasion risk and mitigation of medfly in agricultural landscapes requires knowledge of its population structure and dispersal patterns. Here, estimates of dispersal ability are provided in medfly from South Africa at three spatial scales using molecular approaches. Individuals were genotyped at 11 polymorphic microsatellite loci and a subset of individuals were also sequenced for the mitochondrial cytochrome oxidase subunit I gene. Our results show that South African medfly populations are generally characterized by high levels of genetic diversity and limited population differentiation at all spatial scales. This suggests high levels of gene flow among sampling locations. However, natural dispersal in *C. capitata* has been shown to rarely exceed 10 km. Therefore, documented levels of high gene flow in the present study, even between distant populations (>1600 km), are likely the result of human-mediated dispersal or at least some form of long-distance jump dispersal. These findings may have broad applicability to other global fruit production areas and have significant implications for ongoing pest management practices, such as the sterile insect technique.

## Introduction

Through globalization and increased economic trade, species are frequently transported outside of their natural ranges [1]. For an introduced species to become established and ultimately invasive in new environments, a number of barriers need to be overcome (see e.g. [2]). The impact of invasive species can be wide-ranging, from direct impacts on natural biodiversity and resources to affecting human well-being and agriculture.

A case in hand concerns the Mediterranean fruit fly (medfly), *Ceratitis capitata* (Weidemann) (Diptera: Tephritidae), one of the economically costly pest species worldwide [3], [4]. This species has, through fruit production and associated trade-related transport [5], spread from its native Afrotropical range (following [6]) to several of the main fruit-producing regions across the world [7]. Although somewhat contentious, the geographic extent of the historical (native) range of medfly is now assumed to be Afrotropical [6] but the exact range remains uncertain. The first confirmed presence of medfly in the Western Cape Province, South Africa dates to before the end of the nineteenth century [8], [9], and the species is currently widespread throughout South Africa [10]. Several factors may contribute to the successful and wide-spread establishment of *C. capitata* here and elsewhere. Amongst these are the species’ polyphagous life-history [6], [11], short development time, and high population reproductive potential [12]. *Ceratitis capitata* may also have a broader climate niche compared to its congeners [13], [14].

Current management of medfly populations in South Africa predominantly relies on the use of insecticides, including bait application technique (food baits combined with pesticide), bait stations (food bait and pesticide placed in container or trap) and full-cover sprays [15]. Insecticides are problematic not only for human health but also detrimental to the environment. Consequently, more effective and environmentally-friendly techniques are becoming increasingly sought after. Foremost amongst these is the Sterile Insect Technique (SIT) [16] which involves the release of mass-reared sterile males that mate with wild females thereby decreasing population numbers to a threshold from which populations are unable to recover [17]. SIT is currently used in parts of South Africa (Western Cape) with varying success and limited control of medfly [18]. The successful implementation of SIT relies heavily on information regarding the movement of individuals as well as the effective population sizes for different populations and regions [19]. Traditionally, direct methods, including mark-and-recapture studies, were employed to provide this information. However, recent studies have indicated that such methods may significantly under-estimate population size and migration (see e.g. [20], [21]).

Accurate information regarding population structure and movement of individuals is crucial in ensuring successful implementation of pest-control strategies. For example, incorrect estimates of the minimum size for area-wide pest management could result in a failed attempt and, given the cost involved, funding may not be available to complete or re-start eradication or control programmes [22]. As such, indirect methods such as gene flow estimated from molecular information are increasingly being used (reviewed in e.g. [23]–[25]). Furthermore, an understanding of the population structure allows inferences about movement patterns and neighbourhood sizes (see e.g. [20], [26]–[31]). While it is clear that medfly can have population genetic structure on large geographic scales [3], [27], it is presently unknown at what spatial scale this pattern breaks down or in fact, whether the spatial patterns is dependent on climate or various environmental factors. To address these questions we use a combination of molecular sequence (mitochondrial cytochrome oxidase subunit I) and microsatellite data to estimate population genetic parameters at different spatial scales. The mutation rate of mitochondrial DNA markers are slower than that of microsatellites and can therefore be used to infer historical rather than contemporary (microsatellites) processes that shape evolutionary processes [32]. The null hypotheses we test is that South African populations are equally connected and that there is no population differentiation. We discuss these results in the context of dispersal and pest management.

## Materials and Methods

### Sampling Sites and Fly Collection

**Figure 1:**
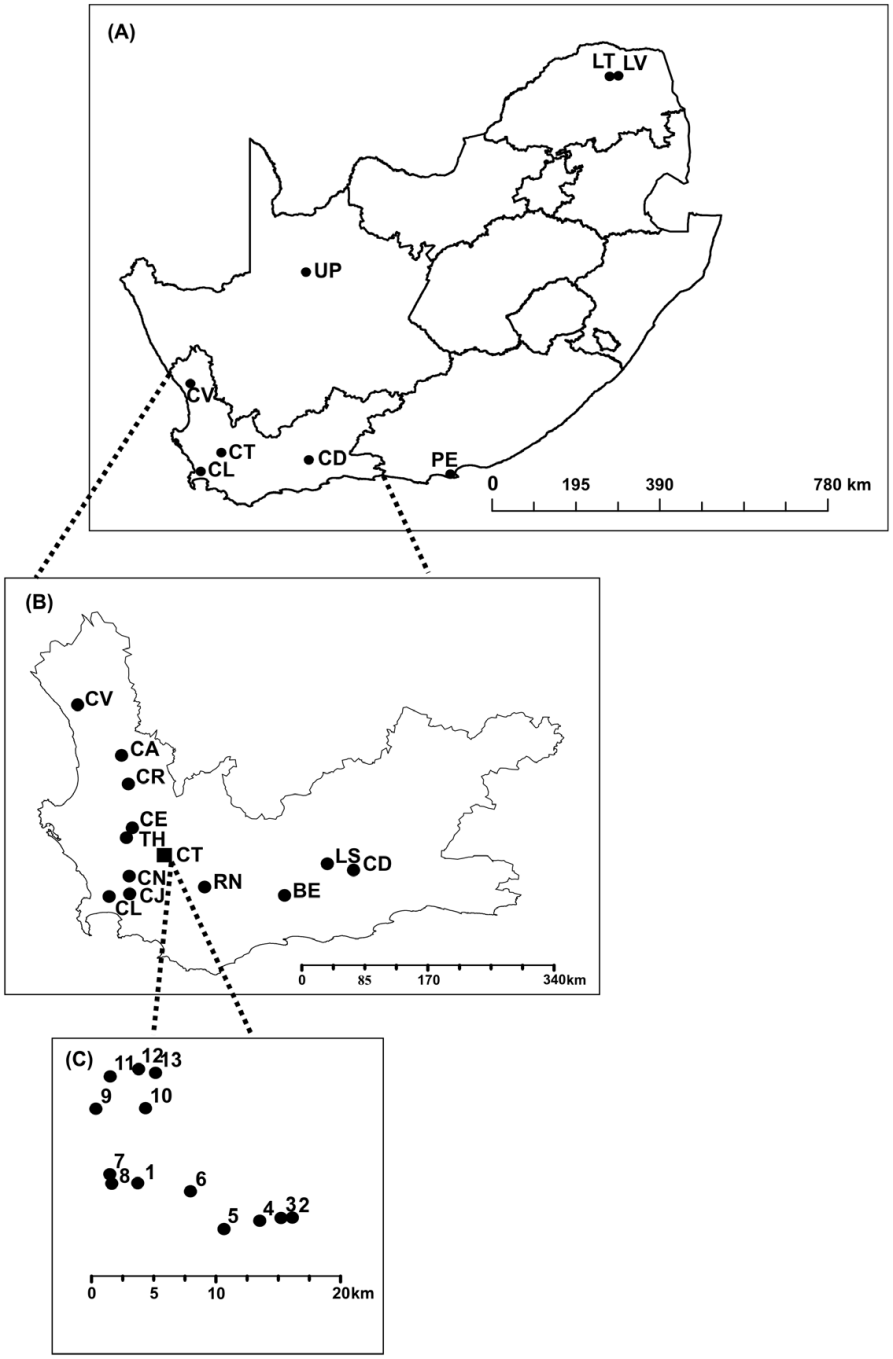
Sampling sites for *Ceratitis capitata* in (A) South Africa, (B) the Western Cape and (C) the Ceres valley. Locations include (CV, Lutzville; CA, Clanwilliam; CR, Citrusdal; CE, Porterville; TH, Tulbagh; CT, Ceres; CN, Wellington, CJ, Simondium; CL, Stellenbosch; RN, Robertson; BE,Barrydale; LS, Ladismith; CD, Calitzdorp; PE, Port Elizabeth; UP, Upington; LV, Levubu; LT, Makhado).

Our sampling regime aimed to capture genetic diversity at three different spatial scales. *Ceratitis capitata* individuals were collected from eight locations in South Africa (broad scale sampling, N = 198 individuals), 13 locations in the Western Cape (regional scale sampling, N = 385) and 13 locations in the Ceres valley (fine scale sampling, N = 382) (Fig. 1, Table 1). Given the nature of our experimental design which aimed to sample medfly in sites currently occupied, sampling focused on the Western Cape as it is an agricultural production area. To ensure spatial homogeneity of sampling effort in our broad scale analyses (South Africa), four locations chosen at random from the Western Cape were included. To ensure that the selection of locations did not have a significant effect on the spatial structure, analyses were repeated several times with a different set of randomly chosen locations; the results remained the same irrespective of the sampling localities included. We therefore report results from only one of these data sets.

**Table 1:** The locations of *Ceratitis capitata* sampling in the Ceres valley, the Western Cape and South Africa with sample size (N), average number of alleles (*N*_A_), number of private alleles (*N*_AP_), allelic richness (*A*_R_, based on a minimum of 3 individuals), expected (*H*_E_) and observed (*H*_O_) heterozygosity (± = standard error) and the inbreeding coefficient (*F*_IS_).

Location	ID	N	*N*_A_	*N*_AP_	*A*_R_	*H*_E_	*H*_O_	*F*_IS_
*Fine scale (Ceres valley)*								
Ceres 1	CT1	30	11.182	4	4.100	0.807±0.102	0.671±0.135	0.185
Ceres 2	CT2	30	11.455	2	4.127	0.812±0.087	0.634±0.184	0.236
Ceres 3	CT3	30	11.182	2	4.099	0.805±0.109	0.662±0.179	0.194
Ceres 4	CT4	30	10.545	1	4.064	0.803±0.100	0.634±0.215	0.227
Ceres 5	CT5	29	10.273	2	3.991	0.793±0.095	0.654±0.192	0.193
Ceres 6	CT6	29	11.182	3	4.147	0.816±0.079	0.660±0.167	0.209
Ceres 7	CT7	25	10.000	0	4.076	0.798±0.097	0.643±0.171	0.215
Ceres 8	CT8	30	10.273	2	4.087	0.808±0.096	0.656±0.127	0.205
Ceres 9	CT9	30	10.545	2	4.127	0.813±0.099	0.665±0.155	0.199
Ceres 10	CT10	29	10.727	3	4.050	0.800±0.087	0.665±0.176	0.187
Ceres 11	CT11	30	10.545	2	4.074	0.808±0.081	0.651±0.168	0.211
Ceres 12	CT12	30	10.364	1	4.117	0.811±0.108	0.617±0.144	0.256
Ceres 13	CT13	30	10.273	3	4.004	0.797±0.094	0.638±0.182	0.217
Total		382	10.657	27	4.082	0.805	0.650	0.210
*Regional scale (Western Cape)*								
Barrydale	BE	26	9.727	1	4.111	0.812±0.096	0.658±0.215	0.209
Calitzdorp	CD	29	11.455	2	4.210	0.822±0.090	0.632±0.216	0.248
Ceres	CT	30	10.455	2	3.991	0.808±0.096	0.658±0.124	0.203
Citrusdal	CR	30	10.182	0	3.958	0.788±0.119	0.593±0.201	0.264
Clanwilliam	CA	30	10.273	3	3.969	0.793±0.097	0.628±0.168	0.225
Ladismith	LS	30	10.545	3	4.030	0.803±0.089	0.672±0.174	0.18
Lutzville	CV	30	9.909	0	3.950	0.793±0.088	0.697±0.198	0.138
Porterville	CE	30	11.364	2	4.115	0.808±0.101	0.640±0.199	0.226
Robertson	RN	30	10.636	2	4.041	0.802±0.100	0.634±0.180	0.227
Simondium	CJ	30	10.364	3	4.036	0.802±0.096	0.632±0.197	0.228
Stellenbosch	CL	30	10.727	4	4.084	0.810±0.088	0.641±0.189	0.225
Tulbagh	TH	30	10.909	4	4.046	0.802±0.104	0.637±0.185	0.222
Wellington	CN	30	10.818	3	4.103	0.805±0.104	0.675±0.217	0.179
Total		385	10.566	29	4.050	0.803	0.646	0.213
*Broad scale (South Africa)*								
Calitzdorp	CD	29	11.455	8	4.210	0.822±0.090	0.632±0.216	0.248
Ceres	CT	30	10.455	3	3.991	0.808±0.096	0.658±0.124	0.203
Levubu	LV	30	11.364	15	4.068	0.793±0.120	0.576±0.183	0.29
Makhado	LT	13	8.636	3	4.177	0.794±0.124	0.585±0.227	0.302
Lutzville	CV	30	9.909	4	3.950	0.793±0.088	0.697±0.198	0.138
Port Elizabeth	PE	6	4.727	1	3.443	0.630±0.200	0.530±0.277	0.26
Stellenbosch	CL	30	10.727	7	4.084	0.810±0.088	0.641±0.189	0.225
Upington	UP	30	9.818	4	3.859	0.777±0.102	0.610±0.199	0.233
Total		198	9.636	45	3.973	0.778	0.616	0.237

Bucket traps (Chempac, Paarl, South Africa) were set up in fruit orchards and geo-referenced using a hand-held GPS. Traps were dry baited with a three-component attractant Biolure 3C (Chempac) consisting of putrecine, ammonium acetate and trimethylamine (attractiveness <30 m; [15]). Traps were collected every two weeks and flies were transferred to absolute ethanol for storage. Flies were identified and sexed under a stereomicroscope in the laboratory. To ensure that flies included in the study were not part of a SIT release (and, as such, not a true reflection of the wild populations), all specimens were inspected under UV light and SIT flies were discarded. Pupae from the SIT program are covered in fluorescent dye before release that accumulates in the head suture during emergence and are thus easily identified under a UV light [18]. DNA was extracted from whole flies using a DNeasy® tissue kit (QIAGEN Inc.).

### Mitochondrial DNA Amplification and Sequencing

For mitochondrial DNA, a 782-bp segment of the cytochrome oxidase subunit I (COI) gene was targeted for ten randomly selected individuals per sampling location where possible. This resulted in a sequence data set of 125 individuals from the Western Cape (Table 2) and 74 individuals from across South Africa (Table 2). The primers C1-J-2183 and TL2-N-3014 [33] were used for amplification following standard procedures for medfly [34]. Sequencing reactions were performed using BigDye chemistry (Applied Biosystems, Foster City, California, USA) and analyzed on an ABI 3170 automated sequencer (Applied Biosystems) (Genbank accession numbers: JX855840- JX855921).

**Table 2:** Sampling locations of *Ceratitis capitata* in Ceres, the Western Cape and South Africa; and genetic diversity indices for the mitochondrial COI gene with N, sample size; N*_h_*, the number of haplotypes; *h,* the haplotype diversity; *π*, nucleotide diversity, Fu’s F-statistic with corresponding significance value (p) (± = standard error) and the identities of the haplotypes (Genbank accession numbers: JX855840- JX855921).

Location	ID	N	N*_h_*	*h*	*π*	Fu’s FS	p	Haplotypes
*Regional scale (Western Cape)*								
Barrydale	BE	10	8	0.956±0.059	0.007±0.004	−1.760	0.130	2Cc1, Cc2, 2Cc3, Cc4, Cc5, Cc6, Cc7, Cc8
Calitzdorp	CD	10	10	1.000±0.045	0.007±0.004	−5.364	0.001	Cc5, Cc7, Cc11, Cc14, Cc15, Cc16, Cc17, Cc18, Cc19, Cc20
Ceres	CT	9	8	0.933±0.077	0.007±0.004	−1.595	0.165	Cc1, Cc7, Cc29, Cc38, Cc47, 2Cc48, Cc49, Cc50
Citrusdal	CR	10	8	0.933±0.077	0.009±0.005	−10181	0.227	Cc7, Cc9, Cc38, Cc42, 3Cc43, Cc44, Cc45, Cc46
Clanwilliam	CA	10	7	0.867±0.107	0.004±0.002	−2.178	0.065	Cc3, Cc7, 4Cc9, Cc10, Cc11, Cc12, Cc13
Ladismith	LS	9	9	1.000±0.052	0.008±0.005	−4.279	0.009	Cc11, Cc26, Cc31, Cc40, Cc56, Cc57, Cc58, Cc59, Cc60
Lutzville	CV	10	8	0.956±0.060	0.007±0.004	−1.770	0.138	Cc9, Cc29, Cc44, Cc51, 2Cc52, 2Cc53, Cc54, Cc55
Porterville	CE	10	10	1.000±0.045	0.007±0.004	−5.520	0.003	Cc9, Cc11, Cc21, Cc22, Cc23, Cc24, Cc25, Cc26, Cc27, Cc28
Robertson	RN	10	9	0.978±0.054	0.006±0.004	−3.604	0.023	Cc7, Cc8, Cc11, 2Cc12, Cc15, Cc38, Cc61, Cc62, Cc63
Simondium	CJ	10	9	0.978±0.054	0.008±0.004	−3.006	0.047	Cc7, Cc9, Cc11, Cc15, Cc26, 2Cc29, Cc30, Cc31, Cc32
Stellenbosch	CL	10	9	0.978±0.054	0.006±0.003	−4.031	0.004	Cc9, 2Cc11, Cc12, Cc25, Cc33, Cc34, Cc35, Cc36, Cc37
Tulbagh	TH	8	7	0.964±0.077	0.004±0.003	−2.928	0.020	Cc3, Cc7, Cc9, Cc59, Cc64, 2Cc65, Cc66, Cc67
Wellington	CN	9	8	0.972±0.064	0.005±0.003	−3.427	0.016	2Cc7, Cc9, Cc12, Cc29, Cc38, Cc39, Cc40, Cc41
Total		125	67	0.980±0.005	0.007±0.004	−25.310	0	
*Broad scale (South Africa)*								
Calitzdorp	CD	10	10	1.000±0.045	0.007±0.004	−5.364	0.004	Cc5, Cc7, Cc11, Cc14, Cc15, Cc16, Cc17, Cc18, Cc19, Cc20
Ceres	CT	9	8	0.933±0.077	0.007±0.004	−1.595	0.166	Cc1, Cc7, Cc29, Cc38, Cc47, 2Cc48, Cc49, Cc50
Levubu	LV	10	9	0.978±0.054	0.008±0.005	−2.751	0.057	Cc12, Cc56, Cc70, Cc76, Cc77, 2Cc78, Cc79, Cc80, Cc81
Makhado	LT	10	8	0.956±0.059	0.007±0.004	−1.665	0.146	Cc14, 2Cc26, 2Cc38, Cc71, Cc72, Cc73, Cc74, Cc75
Lutzville	CV	10	8	0.956±0.059	0.007±0.004	−1.848	0.125	Cc9, Cc29, Cc44, Cc51, 2Cc52, 2Cc53, Cc54, Cc55
Port Elizabeth	PE	5	4	0.900±0.161	0.007±0.004	0.552	0.529	Cc22, Cc54, 2Cc56, Cc82
Stellenbosch	CL	10	9	0.978±0.054	0.006±0.003	−4.067	0.010	Cc9, 2Cc11, Cc12, Cc25, Cc33, Cc34, Cc35, Cc36, Cc37
Upington	UP	10	6	0.889±0.075	0.007±0.004	0.491	0.581	Cc7, 2Cc55, 2Cc68, 3Cc69, Cc70
Total		74	50	0.990±0.004	0.007±0.004	−25.342	0	

### Mitochondrial DNA Analysis

DNA sequences were aligned and edited in GENEIOUS Pro™ 5.0 software (Biomatters Ltd, New Zealand). We calculated the number of haplotypes (*N*_h_), haplotype diversity (*h*) and nucleotide diversity (*π*) [35] (ARLEQUIN v3.5.1.2; [36]). To test for selective neutrality we used Fu’s F-statistic [37] (ARLEQUIN v3.5.1.2). A parsimony haplotype network was constructed to investigate the relationship between COI haplotypes with a 95% connection limit (TCS v1.21; [38]).

Overall (i.e. all sampling locations considered together) and population pairwise *Φ*_ST_ values were calculated using 1000 permutations (ARLEQUIN v3.5.2.1). We tested for isolation by distance (IBD) using a Mantel test [39] with 1000 permutations by testing for a correlation between genetic distance and geographic distance (ARLEQUIN v3.5.2.1). The minimum straight line distance (i.e. as-the-crow-flies) between the GPS coordinates of sampling sites were taken as the geographic distance.

### Microsatellite Genotyping

Specimens were genotyped for 12 microsatellite markers obtained from previously published studies [40]–[42] (see Table S1). Forward primers were 5′-labelled with one of four fluorophores (6-FAM, HEX, VIC or NED) and microsatellite loci were pooled for amplification if there was no signal inhibition during amplification (See Table S1 for further details). Samples were genotyped on an ABI 3130 Automated Sequencer (Applied Biosystems) and alleles were scored using GENEMAPPER v3.7 (Applied Biosystems). A positive control was included to verify that all plates were read consistently.

### Microsatellite DNA Analysis

Microsatellite loci were tested for departures from Hardy-Weinberg equilibrium (HWE) and linkage disequilibrium using 10 000 permutations (GENEPOP; [43], [44]). Significance levels were adjusted using False Discovery Rates ([45]; QVALUE; [46]). The marker Medflymic88 was found to have limited polymorphism and was subsequently excluded from further analyses (Table S1).

Levels of genetic diversity were assessed by computing basic statistics for 11 microsatellite loci. The average number of alleles (*N*_A_), expected heterozygosity (*H*_E_, expected allele frequencies given under Hardy-Weinberg equilibrium) and observed heterozygosity (*H*_O_, actual heterozygosity measured in a population) were calculated for each microsatellite locus and for each location (GENETIX v4.05.2; [47]; GenAlEx 6.4; [48]). Allelic richness (*A*_R_), which is a measure of genetic diversity independent of sample size, was calculated in FSTAT v2.9.3.2 [49]. The inbreeding coefficient (*F*_IS_) was calculated in GENETIX v4.05.2 with 10 000 permutations to assess deviations from the null hypothesis of no inbreeding (*F*_IS_ = 0).

To assess the degree of population differentiation (at broad-, regional- and fine- spatial scales), we used three complementary approaches: *F*_ST_ and two Bayesian clustering methods, one with and one without prior spatial information. These analyses were performed on the three datasets independently. First, pairwise *F*_ST_ values (a measure of the genetic variance in a subpopulation compared to the total genetic variance in the entire population) and overall *F*_ST_ values were calculated in ARLEQUIN v3.5.1.2 using 10 000 permutations [36]. Second, STRUCTURE v2.3.3 [50], [51] was run to assign the multilocus genotypes of individuals to populations without any prior spatial information. To estimate the number of clusters (*K*), we ran 10 independent runs for each *K* value varying between 1 and 13. A burn-in period of 1 000 000 followed by 1 000 000 Markov Chain Monte Carlo (MCMC) permutations was run to allow statistical parameters to reach stability and gave consistent results over the 10 independent runs. To determine the true number of populations (*K*) in the dataset, the method described by Evanno et al. (2005) [52] was used. The STRUCTURE output was visualized using DISTRUCT v1.1 [53]. Thirdly, TESS v2.3 [54], [55], which has an option to implement an admixture model that uses spatial coordinates of populations as prior information, was used to detect spatial genetic structure. Results from STRUCTURE and TESS were concordant; therefore we only discuss results from STRUCTURE (for TESS results see Fig. S2). Spatial genetic structure was investigated using two different approaches. First, a Mantel test was used to assess the significance of the association between geographic and genetic distance (IBD) (implemented in ARLEQUIN v3.5.1.2). Secondly, in SPAGEDI v1.3 [56] we characterised the spatial genetic structure of sampled locations using global *F*-statistics. The standard error of the *F*_ST_ computation was estimated using 10 000 permutations. The results were visualized by plotting the *F*_ST_/(1−*F*_ST_) values against the geographic distance between sampled locations.

## Results

### Mitochondrial DNA Analysis

#### Genetic structure within the Western Cape (regional spatial scale)

We identified 67 distinct haplotypes for the 125 individuals included at the regional spatial scale. Haplotype diversity was notably high with an average value for the region of 0.980 (Table 2). Overall nucleotide diversity was 0.007 (which is comparable to other *Ceratitis* spp.; see e.g. [57]) (Table 2). Fu’s F-statistic was negative and significant (FS = −25.310; p<0.001) indicating a deviation from equilibrium.

**Figure 2:**
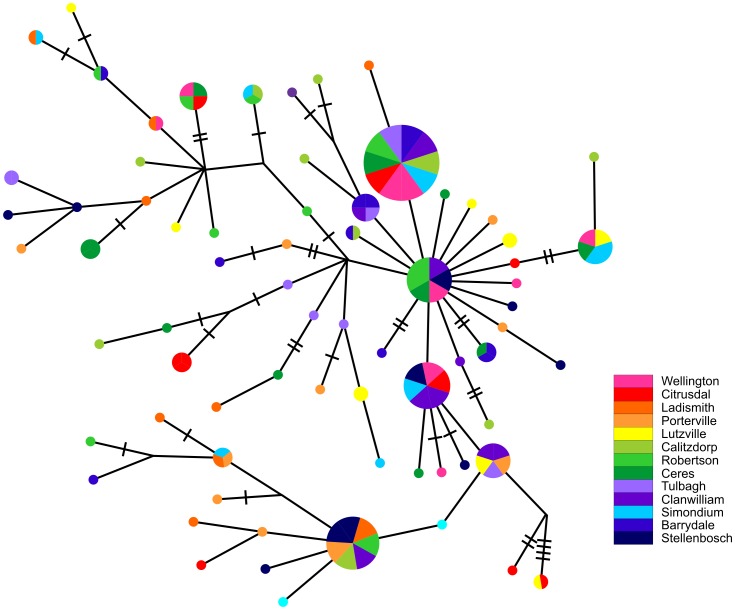
The parsimony haplotype network for *Ceratitis capitata* in the Western Cape (regional scale). The size of the pie charts is representative of the number of individuals that possess that haplotype. The small pie charts show haplotypes with a frequency of one individual. Every connecting line represents a mutational step of one between the different haplotypes. The perpendicular lines indicate additional mutational steps.

The haplotype network showed no explicit spatial pattern of genetic variation across the Western Cape (Fig. 2). Rather, the spatial population structure appeared almost random. Although variation was overwhelmingly partitioned within sampling locations (only 2.9% of the variation was accounted for by the between-site component), we found that the overall *Φ*_ST_ value was significant (*Φ*_ST_ = 0.03, p = 0.01). Pairwise *Φ*_ST_ comparisons between sampling sites were mostly non-significant except for the Tulbagh sampling location which was significantly differentiated from four other sampling localities (Simondium, Citrusdal, Ladismith and Robertson) (see Table S2). A closer inspection revealed the presence of four unique haplotypes in this sampling location. Excluding the Tulbagh sampling location from the AMOVA returned a marginally non-significant partitioning of genetic variation (*Φ*_ST_ = 0.022, p = 0.051). No correlation between genetic and geographic distances were found (regression coefficient = −0.000086, p = 0.927) indicating the absence of isolation by distance.

#### Genetic structure across South Africa (broad spatial scale)

We identified 50 haplotypes for the 75 individuals included from across South Africa. Haplotype diversity was high with an average value for South Africa of 0.990 (Table 2) with nucleotide diversity being 0.007 (comparable to other *Ceratitis* spp.; see e.g. [57]) (Table 2). Fu’s F-statistic was highly negative and significant (FS = −25.342; p<0.001) indicating a deviation from equilibrium.

**Figure 3:**
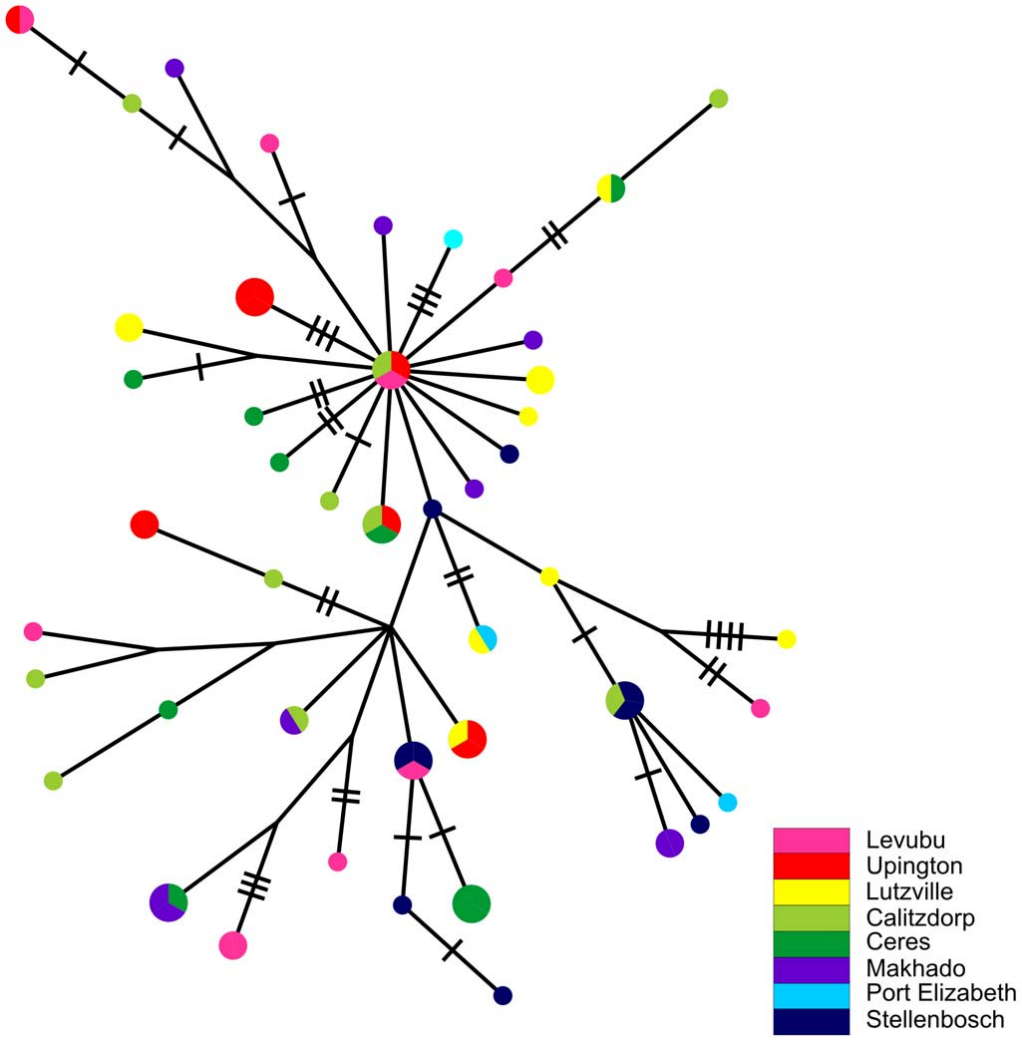
The parsimony haplotype network for *Ceratitis capitata* in South Africa (broad scale). The size of the pie charts is representative of the number of individuals that possess that haplotype. The small pie charts show haplotypes with a frequency of one individual. Every connecting line represents a mutational step of one between the different haplotypes. The perpendicular lines indicate additional mutational steps.

The haplotype network showed no clear spatial pattern of genetic variation in South Africa (Fig. 3). Variation was overwhelmingly partitioned within localities (*Φ*_ST_ = 0.028; p = 0.04). Pairwise *Φ*_ST_ values between sampling sites were small and non-significant. The only significant comparison was between Stellenbosch and Makhado, situated more than 1 600 km apart (Table S3). No correlation between genetic and geographic distances was found (regression coefficient = −0.00001, p = 0.789) indicating the absence of isolation by distance.

### Microsatellite DNA Analysis

No linkage disequilibrium was observed among the 11 polymorphic microsatellite markers. All sampling locations deviated from HWE (genotype frequencies differ from ideal population which are characterized by random mating, no drift, mutation or migration) with relatively high levels of inbreeding indicative of non-random mating within sampling locations (Table 1).

#### Genetic structure within the Ceres Valley (fine spatial scale)

From the Ceres Valley an average of 10.657 alleles were found with all of the sampling localities having private alleles (*N*_AP_) except for Ceres 7 (Table 1). Genetic diversity, as indicated by mean expected heterozygosity (*H*_E_), was 0.805 (Table 1). Allelic richness (*A*_R_) ranged from 3.991 (Ceres 5) to 4.147 (Ceres 6) (mean = 4.082, based on minimum of 3 individuals; Table 1) and the inbreeding coefficient (*F_I_*_S_) ranged between 0.185 (Ceres 1) and 0.256 (Ceres 12) (Table 1).

**Figure 4:**
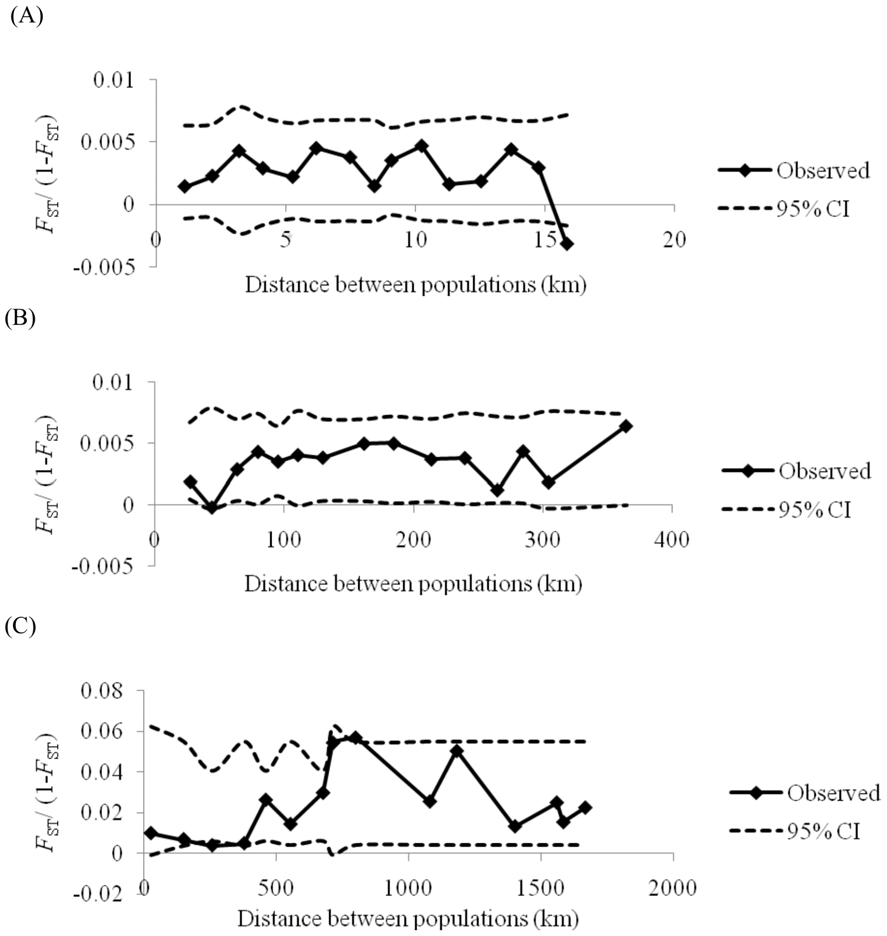
The spatial genetic structure of *Ceratitis capitata* in (A) the Ceres valley, (B) the Western Cape and (C) South Africa. The solid line represents the mean of the multilocus pairwise *F*_ST_/(1−*F*_ST_) values within each distance class and the dashed lines represent the 95% confidence intervals of the null distributions obtained from 1000 random permutations.

The overall *F*_ST_ value was 0.004 (p = 0.182), indicating no significant population differentiation. All of the pairwise *F*_ST_ values (quantification of genetic structure between populations) for sampling localities in the Ceres valley were not significant after corrections for multiple testing using False Discovery Rates. Both the Mantel test and the global *F*-statistics did not indicate any pattern of isolation by distance (r = 0.000002, p = 0.45; Fig. 4A).

The investigation of population differentiation in the Ceres valley using a Bayesian clustering approach in STRUCTURE (Fig. S1) showed a lack of population differentiation. Evanno’s method for estimating the optimal number of clusters (*K*) cannot calculate a Δ*K* value at *K* = 1, as it uses the second order rate of change. Examination of the log of the posterior probability of the data [ln P(D)] for each *K* value revealed the highest ln P(D) value at *K* = 1, an indication of the lack of population differentiation.

#### Genetic structure within the Western Cape (regional spatial scale)

An average of 10.566 alleles was detected with all of the sampling localities having private alleles (*N*_AP_) except for Lutzville and Citrusdal (Table 1). Average genetic diversity (*H*_E_) for *C. capitata* in the Western Cape was high (0.803) (Table 1). Allelic richness (*A*_R_) ranged from 3.950 (Lutzville) to 4.210 (Calitzdorp) (mean = 4.050, based on a minimum of 3 individuals; Table 1) and the inbreeding coefficient (*F_I_*_S_) ranged between 0.138 (Lutzville) and 0.264 (Citrusdal) (Table 1).

Similar to the mtDNA findings, almost all of the pairwise *F*_ST_ comparisons were not significant after False Discovery Rate corrections (Table S4). The highest level of genetic differentiation was between Tulbagh and Citrusdal (*F*_ST_ = 0.019). The overall *F*_ST_ value of 0.006 (p = 0.002) indicated weak but significant population differentiation possibly due to the few significant pairwise comparisons. Results from STRUCTURE for the Western Cape (Fig. S1) indicated no population differentiation and no isolation by distance (Mantel test: r = 0.000011, p = 0.128; see also Fig. 4B for global *F*-statistics).

We identified an average of 9.636 alleles with all of the sampling locations having private alleles (*N*_AP_) (Table 1). Genetic diversity (*H*_E_) for *C. capitata* in South Africa was high (0.778) (Table 1) and the mean value of Allelic richness (*A*_R_) was 3.973 (based on a minimum of 3 individuals; Table 1). The inbreeding coefficient (*F_I_*_S_) ranged between 0.138 (Lutzville) and 0.302 (Makhado) (Table 1).

Pairwise *F*_ST_ values were used to quantify the genetic structure between sampling locations and some of the comparisons were not significant after FDR corrections (Table S5). The highest level of genetic differentiation (*F*_ST_ = 0.083) was between Makhado and Port Elizabeth a distance of approximately 1300 km). The overall *F*_ST_ value was 0.021 (p = 0.0001), which indicated weak but significant population differentiation possibly due to the significant pairwise comparisons between a few localities. STRUCTURE results (Fig. S1) showed a lack of population differentiation. Neither the Mantel test nor global *F*-statistics indicated any pattern of isolation by distance (r = 0.000002, p = 0.269; Fig. 4C).

## Discussion

In this study we characterized the genetic diversity, population genetic structure and population connectivity of a major international pest of agriculture, *C. capitata*, at three different spatial scales within South Africa. Several important and perhaps unexpected findings emerged from our results, with significant implications for other regions where this pest occurs.

### Genetic Diversity

Exceptionally high levels of genetic diversity characterize medfly, from the scale of a single fruit-growing valley (Ceres) to its entire range across South Africa. The values reported here are notably higher than those reported for other *Ceratitis* species (*Ceratitis rosa* and *Ceratitis fasciventris*, [58]), but similar to those reported from other countries in Africa (*H*_E_ = 0.750 [27]; *H*_E_ = 0.896 [30]). Furthermore, the diversity values found for *C. capitata* in South Africa are higher than those reported for this species from other invaded regions of the world (Réunion island: *H*_E_ = 0.660; [27]; Australia: *H*_E_ = 0.238–0.606; [30]; the Mediterranean Basin: *H*_E_ = 0.484–0.630; [30]).

Three possible scenarios may account for these higher levels of genetic diversity in medfly. First, it may reflect the importance of propagule pressure and the effect of multiple introductions on their genetic diversity [1], [59]. For example, medfly may spread relatively easily from Kenya to other African countries, including South Africa, perhaps through human-mediated dispersal, thereby invading these countries multiple times. In contrast, the number of introductions to more distant countries such as Australia and Hawaii may be limited because of stricter quarantine control coupled with relative geographic isolation, and as such, result in lower genetic diversity values [60]. Second, although the native range for medfly is considered to be Afrotropical, this is contentious and the natural range may indeed have been broader [6]. Alien/invasive species typically have lower genetic diversity in their introduced ranges compared to their native ranges [60] and, in this respect, may indicate that South Africa falls within the native range. Third, the reported high levels of genetic diversity may be linked with exceptionally large population sizes in South Africa, among other factors (e.g. high mutation rate, see discussions in [61], [62]). This high genetic diversity may contribute to their ability to colonize novel habitats through increased evolutionary potential [63], [64].

Despite high genetic diversity and proposed large population sizes, all locations sampled from South Africa showed slight levels of inbreeding (*F*_IS_ (Ceres) ranged between 0.185–0.260; *F*_IS_ (Western Cape) ranged between 0.138–0.260; *F*_IS_ (South Africa) ranged between 0.138 and 0.302). Microsatellite estimates of *F*_IS_ in medfly have only been reported once previously [17], based on only 36 individuals randomly sampled on the Island of Chios, Greece. It is therefore difficult to place these findings in a global perspective, or evaluate the expected range of *F*_IS_ in medfly. Although inbreeding seems unlikely given the large effective population sizes we report and that our results suggest that populations in South Africa may be expanding (Fu’s F-statistic) (possibly due to an increase in the area under fruit production), this possibility cannot be excluded as medlfy demonstrates lekking behaviour [61]. Specifically, a small percentage of males can account for the majority of matings [65] suggesting non-random gene combination may be characteristic of the species.

### Genetic Structure

In addition to high levels of genetic diversity, we find limited genetic differentiation among sampling locations across all spatial scales in South Africa. Given that the natural dispersal distances of *C. capitata* rarely exceeds 10 km [66], this was unexpected. Although some sampling locations are genetically significantly differentiated, these are mostly confined to single localities (such as Tulbagh) although the majority of sampling localities exhibit private alleles. Bonizzoni et al. (2004) [30] reported genetic homogeneity and a lack of spatial population differentiation in *C. capitata* populations in the coastal regions of Australia from Perth northwards. By contrast, Alaoui et al. (2010) [31] found population genetic structure in Moroccan *C. capitata* populations in endemic Argan forests, predominantly driven by their occurrence at different altitudes. We suggest that the structure of medfly in South Africa may be the result of complex interactions among factors at play at local scales (such as limited dispersal ability and adaptation -genetic and/or physiological- to local environments) and broad spatial scales, such as human-mediated or other forms of long distance dispersal. Indeed in South Africa large parts of the country are essentially climatically unsuitable for medfly and lacking in host plants; however, there are active commercial trade routes linking fruit distribution among these areas.

### Dispersal

Owing to its global pest status, many investigations have focused on quantifying the dispersal ability of *C. capitata*. Although limited genetic structure and higher levels of population connectivity may have been expected for the Ceres valley (maximum distance between sampling localities is 17 km), this was an unexpected finding for our regional and broad scale investigation as sampling sites are geographically more distant, being up to 1600 km apart. Sharp and Chambers (1976) [67] showed that *C. capitata* can fly a maximum distance of 7–8 km, whereas most individuals only flew between 1–3 km within 2–3 hours. Similarly, Meats and Smallridge (2007) [66] indicated dispersal in *C. capitata* of between 0.5–9.5 km through natural dispersal (mainly flight), but that only a very small percentage of individuals are likely to do so (90% of released individuals remain within 400–700 m from release point). Therefore, given their limited dispersal ability based on direct estimates, the gene flow estimates are perhaps higher than might be expected. However, direct estimates are typically measured under natural or semi-natural conditions at a specific time point and may exclude the movement of individuals under extreme conditions, over longer time scales or multiple generations [68]. Therefore, although natural long-distance dispersal events by active flight or passive dispersal on wind are rare, they might nevertheless be frequent enough to cause genetic homogeneity among populations [68], [69].

The distribution of suitable host plants (both wild and cultivated) in South Africa is probably able to facilitate the movement of *C. capitata* using their natural dispersal ability, although these are interspersed with large areas of unfavourable habitat. Therefore, these long distance dispersal events might occur in parallel or in series with human-mediated dispersal which can take place over even longer distances. The most likely vectors of human-mediated dispersal include fruit consignments, the movement of nursery material (ornamental plants, e.g. in the Rosaceae and Liliaceae families) and also the movement of fruit between different locations throughout the country [19], [70], [71]. Regardless of the precise mode of movement, the overall result is potentially increased genetic homogenization.

### Implications for Pest Management Strategies

If these results are assumed to be a reliable indication of movement patterns and population structure in South Africa, one potential implication of this work is that it suggests area-wide pest management should perhaps be undertaken at a broad scale, rather than on a fine scale (eg. farm or valley basis), as is presently the case [72]. Our results therefore imply that the whole of South Africa should potentially be considered a management unit, although this result should be verified further. While managing the whole of South Africa as a single unit may be considered unfeasible, it raises a number of practical issues for current control practise. First, if the whole country is not simultaneously targeted for control, any small-scale, localized control effort is likely to fail owing to high connectivity. It may therefore be worthwhile restricting the movement of fruit produce within the country, or allowing trade movement only after ensuring it is pest-free. Second, it may be important to consider high-risk routes of fruit movement and simply screen or quarantine those consignments most likely to move *C. capitata* around the country in order to eliminate this invasion pathway. Finally, it also suggests that import trade could be contributing to the high gene flow patterns detected (i.e. through propagule pressure). In this respect, it would be worthwhile to assess gene flow into South Africa from other adjacent countries or revisit global estimates of gene flow in medfly to determine if the global pattern previously documented [3], [27] is changing, as this could help identify the reason for such low genetic structure in South Africa. It is increasingly clear, however, that the appropriate management unit should encompass wild host areas, home gardens and multi-owner fruit orchards to prevent *C. capitata* from taking refuge and recolonizing agricultural areas under control [73]. Furthermore, results of the current study can be used in future as a reference point to assess the success of SIT in South Africa. The expectation would be that genetic diversity would decrease as a result of decreasing population size if SIT is successful in suppressing the pest population. The present study is thus useful for better understanding the population structure of *C. capitata*, and in turn, could facilitate improved area-wide pest management programs for sustainable crop production in these and other geographic regions**.**

## Supporting Information

Figure S1 **Analysis of** ***Ceratitis capitata*** **individuals from (A) the Ceres valley, (B) the Western Cape and (C) South Africa using the Bayesian based method implemented in the program STRUCTURE.** Each individual is indicated with a vertical line, the different shades of grey represent the individual’s estimated percentage membership to the *K* clusters. Genetic population structure is shown for *K* = 2.
(PDF)

Figure S2 **Analysis of** ***Ceratitis capitata*** **individuals from (A) the Ceres valley, (B) the Western Cape and (C) South Africa using the Bayesian based method implemented in the program TESS.** Each individual is indicated with a vertical line, the different shades of grey represent the individual’s estimated percentage membership to the *K* clusters. Genetic population structure is shown for *K* = 2.
(PDF)

Table S1 (PDF)

Table S2 (PDF)

Table S3 (PDF)

Table S4 (PDF)

Table S5 (PDF)
