# Supplementary figures and images for: Population Genetics of Ceratitis capitata in South Africa: Implications for Dispersal and Pest Management
Source: PLoS One. 2013 Jan 16;8(1):e54281. doi: 10.1371/journal.pone.0054281 (PMC3547002; doi:10.1371/journal.pone.0054281)

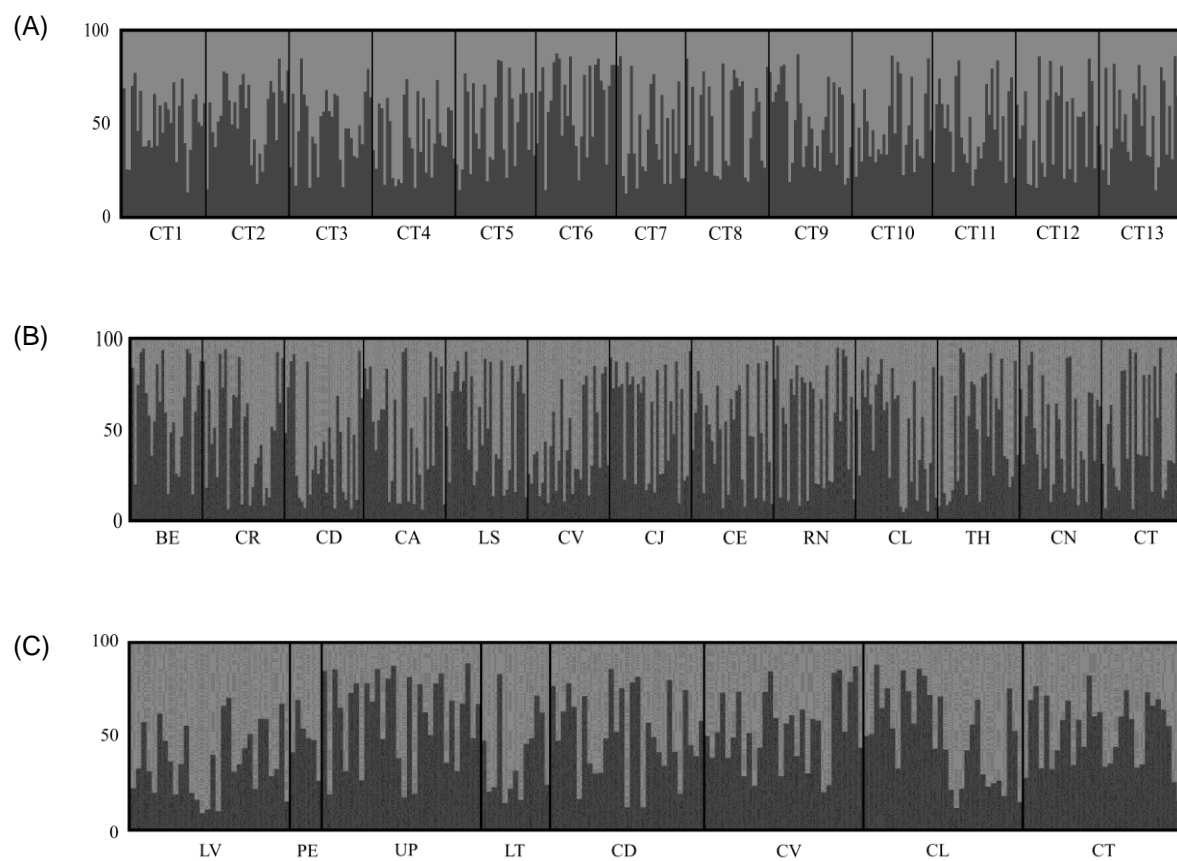

**Figure S1.**

Supplement: Figure S1 — Analysis of Ceratitis capitata individuals from (A) the Ceres valley, (B) the Western Cape and (C) South Africa using the Bayesian based method implemented in the program STRUCTURE. Each individual is indicated with a vertical line, the different shades of grey represent the individual’s estimated percentage membership to the K clusters. Genetic population structure is shown for K = 2. (PDF) [file pone.0054281.s001.pdf]

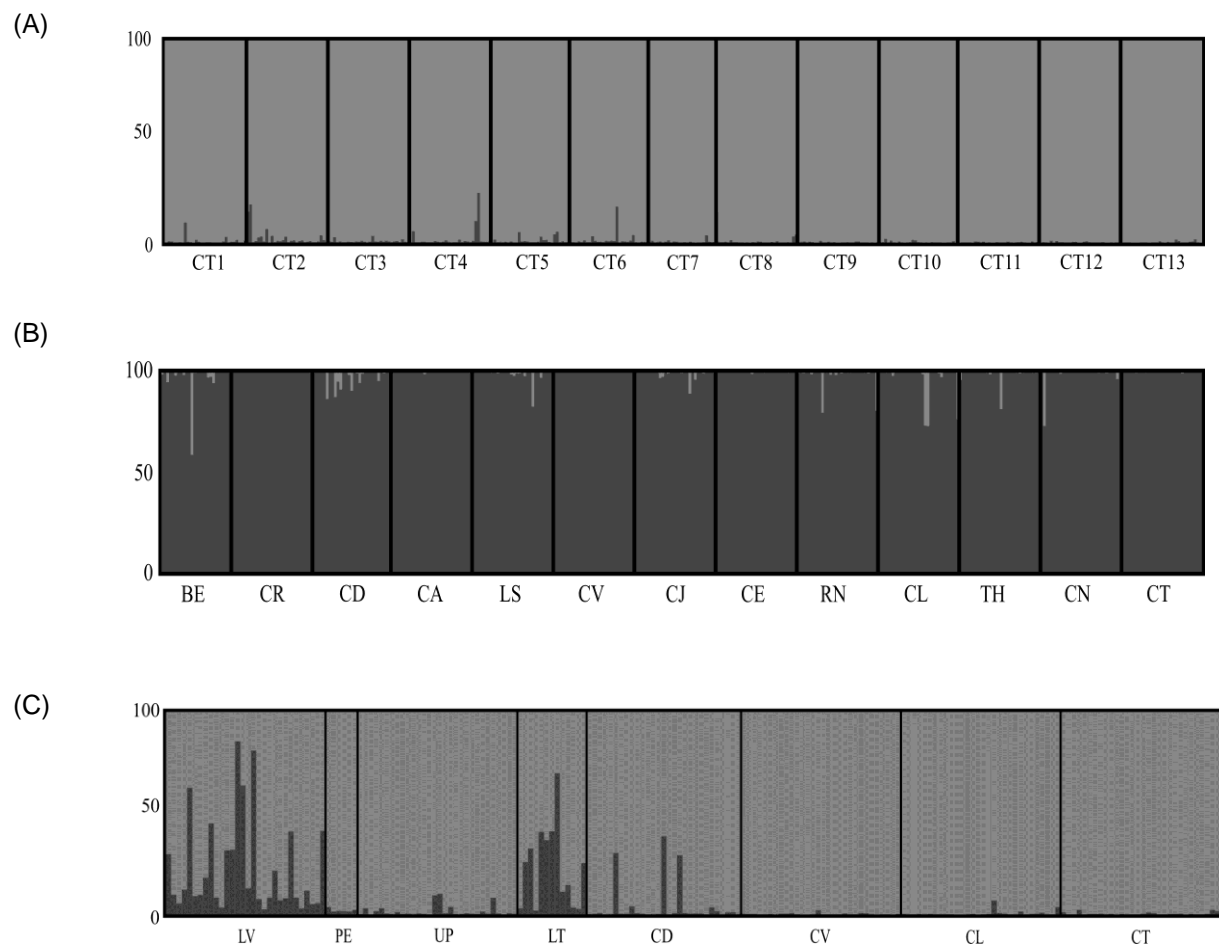

**Figure S2.**

Supplement: Figure S2 — Analysis of Ceratitis capitata individuals from (A) the Ceres valley, (B) the Western Cape and (C) South Africa using the Bayesian based method implemented in the program TESS. Each individual is indicated with a vertical line, the different shades of grey represent the individual’s estimated percentage membership to the K clusters. Genetic population structure is shown for K = 2. (PDF) [file pone.0054281.s002.pdf]
