# Supplementary material for: Population Genetics of Ceratitis capitata in South Africa: Implications for Dispersal and Pest Management
Source: PLoS One. 2013 Jan 16;8(1):e54281. doi: 10.1371/journal.pone.0054281 (PMC3547002; doi:10.1371/journal.pone.0054281)
Supplement: Table S1 — (PDF) [file pone.0054281.s003.pdf]

Table S1.

Locus name	Repeat motif	Multiplex	Label	Size range (bp)	T_A (^{0}C)	N_A	H_O	H_E
Ccmic9[1]	(GA)9TA(GA)5TAGG(GA)2TA(GA)6TAGATA(GA)13	2	VIC	102-167	55	28	0.5572	0.8641
Ccmic6[1]	(TG)18	2	NED	68-117	55	24	0.3691	0.8986
Ccmic8[1]	(TG)2GG(TG)5CG(TG)5	1	PET	111-143	55	13	0.731	0.8191
Ccmic12[1]	(CA)14AA(CA)3AA(CA)3	2	6FAM	71-118	55	22	0.5733	0.8949
Ccmic14[1]	(CA)10CCAA(CA)2	1	VIC	66-87	55	8	0.7709	0.7857
Ccmic3[1]	(TG)11	2	PET	66-93	55	15	0.5401	0.8425
dccap6[2]	(AT)2AG(AT)4(AC)2(AT)2(AC)3	2	6FAM	212-245	55	13	0.4676	0.5732
Medflymic44[3]	(TG)13	1	6FAM	149-202	55	25	0.7514	0.8275
Medflymic96[3]	(TG)11(CAA)3	2	PET	159-237	55	27	0.8517	0.89
Medflymic88[3*]	A8/(GAA)3	1	VIC	179-239	55	2	0	0
Medflymic128[3]	(TG)4TA(TG)14	1	NED	163-220	55	28	0.8719	0.9107
Medflymic25[3]	(TA)9	1	NED	248-295	55	19	0.6412	0.7622

[1] [40]; [2] [41]; [3] [42]

* Medflymic88, not polymorphic, not included in further analysis
