# Supplementary material for: Population Genetics of Ceratitis capitata in South Africa: Implications for Dispersal and Pest Management
Source: PLoS One. 2013 Jan 16;8(1):e54281. doi: 10.1371/journal.pone.0054281 (PMC3547002; doi:10.1371/journal.pone.0054281)
Supplement: Table S2 — (PDF) [file pone.0054281.s004.pdf]

Table S2.

Population	1	2	3	4	5	6	7	8	9	10	11	12	13
1 Barrydale	0												
2 Clanwilliam	0.038	0											
3 Calitzdorp	0.000	0.014	0										
4 Porterville	0.054	0.039	0.052	0.000									
5 Simondium	0.032	0.015	-0.006	-0.031	0.000								
6 Stellenbosch	**0.075***	-0.004	0.055	-0.050	-0.004	0.000							
7 Wellington	-0.015	0.023	-0.010	**0.075***	-0.004	0.079	0.000						
8 Citrusdal	**0.094***	0.066	0.016	0.057	0.031	0.069	0.054	0.000					
9 Ceres	0.022	0.046	0.007	0.054	0.010	0.076	-0.013	0.027	0.000				
10 Lutzville	-0.006	0.023	-0.005	0.059	0.003	**0.056***	-0.030	0.037	-0.007	0.000			
11 Ladismith	0.056	0.022	0.023	-0.065	-0.063	-0.031	0.057	0.027	0.044	0.056	0.000		
12 Robertson	0.031	0.032	-0.020	0.010	-0.026	0.033	-0.020	0.008	0.004	0.011	-0.003	0.000	
13 Tulbagh	0.052	0.101	0.041	0.110	**0.100***	0.099	0.067	**0.1068***	0.046	0.029	**0.136***	**0.090***	0

*Statistical significance at p<0.05
