# Supplementary material for: Population Genetics of Ceratitis capitata in South Africa: Implications for Dispersal and Pest Management
Source: PLoS One. 2013 Jan 16;8(1):e54281. doi: 10.1371/journal.pone.0054281 (PMC3547002; doi:10.1371/journal.pone.0054281)
Supplement: Table S3 — (PDF) [file pone.0054281.s005.pdf]

Table S3.

Population	1	2	3	4	5	6	7	8
1 Calitzdorp	0							
2 Stellenbosch	0.030	0						
3 Ceres	0.007	0.056	0					
4 Lutzville	-0.006	0.045	-0.001	0				
5 Upington	0.032	0.116	0.086	0.057	0			
6 Makhado	-0.018	**0.020***	0.005	0.008	0.079	0		
7 Levubu	-0.014	0.053	-0.016	0.017	0.067	-0.017	0	
8 Port Elizabeth	0.029	-0.003	-0.032	0.027	0.128	-0.005	-0.016	0

*Statistical significance at $p<0.05$
