# Supplementary material for: Population Genetics of Ceratitis capitata in South Africa: Implications for Dispersal and Pest Management
Source: PLoS One. 2013 Jan 16;8(1):e54281. doi: 10.1371/journal.pone.0054281 (PMC3547002; doi:10.1371/journal.pone.0054281)
Supplement: Table S4 — (PDF) [file pone.0054281.s006.pdf]

Table S4.

Population	1	2	3	4	5	6	7	8	9	10	11	12	13
1 Barrydale	0												
2 Citrusdal	0.002	0											
3 Calitzdorp	0.004	0.008	0										
4 Clanwilliam	0.009	0.005	0.008	0									
5 Ladismith	-0.002	0.002	0.000	0.009	0								
6 Lutzville	**0.014***	0.005	0.006	0.005	0.001	0							
7 Simondium	-0.001	0.008	0.005	0.007	-0.002	**0.010***	0						
8 Porterville	0.001	0.002	-0.003	0.002	-0.003	0.001	0.003	0					
9 Robertson	-0.001	0.005	0.005	**0.011***	0.001	**0.014***	0.007	0.005	0				
10 Stellenbosch	0.000	0.009	**0.011***	0.008	0.007	**0.013***	0.006	0.010	0.005	0			
11 Tulbagh	0.004	**0.019***	0.003	**0.016***	0.004	**0.012***	0.007	0.005	**0.014***	0.009	0		
12 Wellington	-0.005	0.006	0.002	**0.012***	-0.004	0.006	0.008	0.001	0.001	0.002	0.005	0	
13 Ceres	0.003	**0.016***	0.003	**0.017***	0.002	**0.013***	0.008	0.003	0.009	**0.016***	0.000	0.001	0

* Statistical significance at $q < 0.05$ after FDR correction
