# Supplementary material for: Population Genetics of Ceratitis capitata in South Africa: Implications for Dispersal and Pest Management
Source: PLoS One. 2013 Jan 16;8(1):e54281. doi: 10.1371/journal.pone.0054281 (PMC3547002; doi:10.1371/journal.pone.0054281)
Supplement: Table S5 — (PDF) [file pone.0054281.s007.pdf]

Table S5.

Population	1	2	3	4	5	6	7	8
1 Levubu	0							
2 Port Elizabeth	**0.064**	0						
3 Upington	**0.020**	**0.075**	0					
4 Louis Trichardt	0.011	**0.083**	0.013	0				
5 Calitzdorp	**0.014**	**0.061**	0.009	0.013	0			
6 Lutzville	**0.024**	**0.081**	**0.026**	**0.019**	0.007	0		
7 Stellenbosch	**0.029**	**0.065**	**0.026**	**0.019**	0.009	**0.015**	0	
8 Ceres	**0.012**	**0.066**	**0.028**	0.011	0.005	**0.017**	**0.016**	0

* Statistical significance at $q < 0.05$ after FDR correction
